# Evaluating compliance to a cardiac rehabilitation program in a private general hospital

**DOI:** 10.1590/S1679-45082013000300004

**Published:** 2013

**Authors:** Vanessa Mair, Ana Paula Breda, Marcos Eduardo Boquembuzo Nunes, Luciana Diniz Nagem Janot de Matos

**Affiliations:** 1 Hospital Israelita Albert Einstein, São Paulo, SP, Brazil

**Keywords:** Rehabilitation, Absenteeism, Questionnaires, Cardiovascular diseases, Exercise

## Abstract

**Objective:**

Identify the primary factors that influenced the participant in our cardiovascular rehabilitation program towards missing their therapy sessions, and to correlate those factors with age, cardiovascular risk, and motivation of our population.

**Methods:**

We conducted a retrospective study with 42 patients (69.15±13.93 years) participating in the cardiac rehabilitation program at a general hospital in São Paulo, through the analysis of two scales applied during the initial evaluation: Cardiac Rehabilitation Barriers Scale and scale of the original provision. We used Spearman correlation to relate them to absenteeism, cardiovascular risk age and duration of cardiac rehabilitation.

**Results:**

The total score of barriers was 31±6 and the mean score of 1.47±0.31. The main barriers subscales were related to “travel/labor dispute” and “personal problems/family.” The percentage of absenteeism was 8.4% in the number of sessions that could be made in the month. The faults and cancellations were positively correlated with cardiovascular risk (p=0.01; r=0.4) and negatively with scale provision of baseline (p=0.03; r=-0.35) and age (p=0.02; r=-0.35).

**Conclusion:**

“Travel/labor dispute”, “personal/ family problems”, and low initial provision are the main factors absenteism in a cardiac rehabilitation program in a general hospital in São Paulo.

## RESUMO

**Objetivo:**

Identificar os principais fatores que influenciaram o participante do nosso programa de reabilitação cardiovascular a faltar em suas terapias e correlacioná-los a idade, risco cardiovascular e motivação de nossa população.

**Métodos:**

Realizou-se um estudo retrospectivo transversal com 42 pacientes (69,15±13,93 anos) participantes do programa de reabilitação cardíaca de um hospital geral particular de São Paulo, por meio da análise de duas escalas: a Escala de Barreiras para Reabilitação Cardíaca e a Escala de Disposição. Utilizou-se a correlação de Spearman para relacioná-las com absenteísmo, risco cardiovascular, idade e tempo de reabilitação cardíaca.

**Resultados:**

A pontuação total da escala de barreiras foi 31±6 e o escore médio foi de 1,47±0,31. As principais barreiras foram relacionadas às subescalas “viagem/conflito de trabalho” e “problemas pessoais/familiares”. A porcentagem de absenteísmo foi 8,4% do número de sessões possíveis de serem realizadas no mês. As faltas e os cancelamentos apresentaram correlação positiva com o risco cardiovascular (p=0,01; r=0,4) e correlações negativas com Escala de Disposição inicial (p=0,03; r=-0,35) e idade (p=0,02; r=-0,35).

**Conclusão:**

“Viagem/conflito de trabalho”, “problemas pessoais/familiares” e baixa disposição inicial foram os principais fatores de absenteísmo em um programa de reabilitação cardíaca de um hospital geral particular de São Paulo.

## INTRODUCTION

Cardiovascular diseases (CVD) are responsible for the highest rates of morbidity and mortality worldwide, according to the World Health Organization (WHO). In Brazil, it is estimated that the rate of mortality for these diseases is 61.9/1 million inhabitants, highlighting as risk factors arterial hypertension, dyslipidemia, diabetes mellitus, sedentarism, obesity, and smoking^(1)^.

Epidemiological data confirm the benefits generated by engagement in regular physical exercise in healthy individuals. In cardiac patients, regular physical training associated with changes in lifestyle have direct and indirect effects on the cardiovascular system, and can improve functional capacity and reduce the possibility of new events and hospitalizations^(2–4)^.

Cardiovascular rehabilitation (CR) is characterized as a non-drug intervention and is defined as activity necessary to ensure the best physical, psychological, and social conditions for cardiac patients, preserving and improving quality of life, and reducing risk factors^(5,6)^. It is recommended especially for patients after myocardial infarction and after coronary artery bypass graft (CABG); recently it has also been suggested for patients with chronic coronary disease, stable heart failure, pre- and post-heart transplant, valvar diseases, and peripheral arterial disease^(3)^.

Even though the recommendation class is I and the level of evidence is A^(7)^, the number of patients who participate in CR programs is extremely low, and there are various reasons for this low participation, including a low percentage of patients effectively referred by the primary physician, travel distance, cost of therapy, and restricted number of services that offer supervised treatment to patients^(8)^.

In the United States, only 10 to 20% of the eligible patients participated in a CR program^(9–11)^, and among the patients referred who initiated the program, 40 to 50% drop out before finishing it^(12)^. It is known that personal, professional, and institutional barriers, as well the motivational level may strongly interfere in participation and compliance^(13–15)^.

In Brazil, there is not much information about this. There is a lack of rehabilitation center data as to the profile of the patients refered, as well as the reasons for non-participation and non-continuity in the programs. The first step towards change of this scenario is to map the difficulties encountered by those patients who are already in treatment.

## OBJECTIVE

The objective of this study was to identify the primary factors that influenced the participant in our cardiovascular rehabilitation program towards missing their therapy sessions, and to correlate those factors with age, cardiovascular risk, and motivation of our population.

## METHODS

A retrospective study previously approved by the Ethics Committee of the hospital (Report EP/Einstein 234,887) conducted with participants of the CR program of the Rehabilitation Center of the *Hospital Israelita Albert Einstein* for at least three months, by means of analyses of data stored in databanks. The model of the CR program at our organization is composed of aerobic and resistance exercises, with sessions that can be held two or three times a week, following national and international CR guidelines^(5,7)^.

The instruments used in evaluation and reevaluation were the Cardiac Rehabilitation Barriers Scale (CRBS) and the Readiness to Change Scale. To verify the patient's cardiovascular risk, the stratification of risk of the American College of Sports Medicine was used, which classifies patients at low, moderate, or high cardiovascular risk for cardiac rehabilitation^(16)^.

To determine the rate of absenteeism, the non-attendance events and cancelations of the patients were added, considering only the data from the last month of the program.

### Cardiac Rehabilitation Barriers Scale

The CRBS was developed in Canada and validated in three languages^(17)^, including Portuguese^(15)^. It was created and is used to assess the barriers to participation and compliance to CR. It is composed of 21 items, divided into 4 subscales, each related to a group of barriers (Appendix 1): perceived needs/healthcare factors, with 9 items; logistic factors, with 5 items; work/time conflicts, with 3 items; and comorbidities/functional status, with 4 items.

The participants were asked to classify their degree of agreement with the items by means of a five-point Likert scale^(18)^ that corresponds to: 1 – strongly disagree; 2 – disagree; 3 – neither agree nor disagree; 4 – agree; and 5 – strongly agree.

The possible total scores – maximum and minimum – were 105 and 21, respectively, in which the higher the score, the greater the number of barriers and vice-versa. To determine the mean scores, the sum of all answers to the CRBS items was made, with posterior division of this result by the total number of questions – 21.

### Readiness to Change Scale

A Readiness to Change Scale^(19)^, described by Prochaska and DiClemente in 1986, was used to identify the motivational stage of the patient, and was applied at two time points (evaluation and reevaluation) (Appendix 2). It is formed by two questions with a 1 to 10 score, where 1 and 2 correspond to the precontemplation or nonprepared phase (score 1); 3 to 5 to the contemplation or insecure phase (score 2); 6 to 8 to the preparation or prepared phase (score 3); and 9 and 10 to the action or changing phase (score 4). According to the final score, one can infer the patient's motivational stage so that there may be a specific recommended approach^(19)^.

### Statistical analysis

The personal characteristics of the patients were described as measures (mean±standard deviation - SD) for quantitative measures, and absolute and relative frequencies for qualitative measures.

Spearman correlations were calculated from the total number of absenteeism over the last month with the total score and mean CRBS, and with the scores of the CRBS subscales. Posteriorly, Spearman's correlation was used between the total number of absenteeism over the last month and age, and with the score generated for the cardiovascular risk and for the Readiness to Change Scale.

ANOVA variance analysis was used with repeated measures to compare the scores among the subscales with the total CRBS score, followed by Bonferroni's multiple comparisons. The Statistical Package for Social Science (SPSS) 15.0™ evaluation version (Chicago, IL, USA) was used.

The level of statistical significance was defined as 5% for all tests (p<0.05).

## RESULTS

Forty-two patients aged between 32 and 93 years (69±14 years) were evaluated, 31 (73.8%) of them men. Tw o of these patients responded to the initial questionnaire but were excluded from the study, since one was referred to the neurology sector before completing the cardiovascular program and the other left the program before the ideal time for completing his reevaluation.

The prevalence of the clinical diagnosis of the sample is shown on table 1.

**Table 1:** Prevalence of clinical diagnosis of the sample

Clinical diagnosis	Sample n (%)
Coronary insufficiency	20 (47.62)
Systolic HF	8 (19.04)
HF with normal ventricular function	2 (4.76)
AF	2 (4.76)
Inappropriate tachycardia	1 (2.38)
Cardiac transplant	1 (2.38)
Diabetic neuropathy	1 (2.38)
Reflex syncope	1 (2.38)
Valve exchange	1 (2.38)
Postpartum myocardiopathy	1 (2.38)
Primary prevention	2 (4.76)

HF: heart failure; AF: atrial fibrillation.

Of the 42 patients assessed, 23 were classified as low cardiovascular risk, 11 as moderate risk, and 8 as high risk, with a mean of 25 months of participation in the cardiac rehabilitation program.

The total number of absences (32) and cancellations (30) during the last month corresponded to 8.4% of the number of sessions possible in one month (380 sessions). Patients that participated twice a week (n=26) had a total of 35 missed sessions and cancellations, and those who went 3 times a week (n=12) had a total sum of 25 missed sessions and cancellations (Table 2).

**Table 2:** Clinical and rehabilitation characteristics

Variable	Mean±SD
Age (years)	69.1±13.9
Time of CR (months)	25.0±25.5
Cardiovascular risk (scores 1-3)	1.6±0.8
Non-attendance and cancellations (sessions)	1.5±1.5

SD: standard deviation; CR: cardiovascular rehabilitation.

The mean total score of answers to the CRBS questionnaire was 31±6, with a mean score of 1.47±0.31.

When the scores were analyzed for each subscale, the highest score was determined for two subscales: “travel/ work conflict” and “personal/family problems” (Table 3). For multiple comparisons, there was a statistically significant mean difference between the total score and these subscales (p<0.05), in which “travel/work conflicts” has greater statistical significance (p<0.01) (Table 3).

**Table 3:** Mean score of the subscales of the Cardiac Rehabilitation Barriers Scale

Variable	Mean±SD
Comorbidity/functional status	1.37±0.39
Perceived needs	1.23±0.39
Personal/family problems	1.44±0.62
Travel/work conflicts	2.77±1.26*
Access	1.29±0.48

ANOVA with repeat measurements;

* p<0.01.

SD: standard deviation.

There was a negative correlation between age and the barrier scale, both when associated with the mean score (p=0.05) and the total score (p=0.05).

Absenteeism correlated positively with cardiovascular risk (p=0.01; r=0.40) and negatively with age (p=0.02; r=-0.35).

Twenty-five patients answered the initial Readiness to Change Scale and 34 patients answered the Readiness to Change Scale at reevaluation. The non-completion of this item occurred due to no participation of the psychologist in all evaluations and reevaluations.

Thirteen patients presented with maximal motivational level (phase corresponding to the action or in process of change – score 4), evaluated by the Readiness to Change Scale at the time of the evaluation, and 12 patients presented with score 3 (phase of preparation or prepared). Twenty-eight patients attained maximum scores for the motivational stage at the reevaluation of 28 patients; 5 presented with score 3; and only 1 patient reported being in the contemplation or insecure phase (score 2).

A negative correlation between the initial Readiness to Change Scale score and the number of absence events was observed, demonstrating that the greater the initial willingness, the lower the number of absences(p=0.03; r=-0.35).

## DISCUSSION

Our results showed findings similar to those of Ghisi et al., for the patients participating in a CR program^(15)^. In our population, the greatest values of the mean score were related to factor 4 - “travel/work conflicts” (score 2.77±1.26), which was not noted by Ghisi et al., since in his findings, factor 1 predominated, i.e., “comorbidities/functional status” (score 1.37±0.39). It is important to point out that the points of our mean limiting score are twice as high as the limiting score reported by Ghisi et al.^(15)^. This may be related to the characteristics of our population, with a high demand related to work and frequent travels, and to our sector, which is comprehensible regarding these needs, encouraging the retention of the patient in the program by flexibility related to the absence events. As long as we are committed to the goals, we do not exclude the patient from the program due to absence for the reasons mentioned above, and we facilitate their presence with frequent rescheduling. Additionally, in patients who are released for supervised training, we advise training adapted to travel, with the intent that they at least not lose the physical conditioning acquired.

The literature reports highlight elderly individuals as those with the highest barriers, since, in general, they are less aware of the benefits of CR, and they have other complaints and comorbidities^(20,21)^. Our findings do not corroborate these statements, since our elderly patients presented with the lowest rates of absenteeism. There is no single explanation for this disagreement, but we could imagine that the high level of instruction of our population might influence these findings. In the study by Ghisi et al., the individuals with high levels of schooling had lower mean scores and greater participation in CR^(15)^. Additionally, our study identified travel and work-related conflicts as the greatest factor linked to non-attendance and cancelations.

During the period evaluated, the patients presented with a total sum of 8.4% absences and cancellations relative to the number of possible sessions to be conducted in one month. The patients who frequented twice a week had a higher number of absences and cancellations relative to those who went three times a week. We can suggest that the patients who agreed to engage in the program three times a week were already committed from the beginning. According to the study carried out by Cooper et al., the patients participating in the CR programs probably believed that it was necessary for their treatment and those who did not participate did not see it as necessary for their management (17.7±2.7 *versus* 16.9±3.0; p=0.029). The study concluded that the barriers for CR may be quantified and differ between participants and non-participants in CR^(16)^. In the same way, we can extrapolate that the commitments of the patients who practice CR three times a week differ from those who do so only twice a week. In addition, considering the negative correlation between the initial Readiness to Change Scale and the number of absences (p=0.03; r=-0.35), it is possible to infer that the patients most motivated from the beginning show better adherence to the program. Studies have shown that belief in CR can have a great influence in adherence to it. It is suggested that there is a classification of specific scales as to the need for and efficacy of the treatment prescribed. Greatest adherence is found in patients that believe in the need for their treatment^(22–24)^. In our study, the patients presented with greater motivation as the study progressed.

Compliance is a critical factor for global management of individual risks for CVD. It forms an interaction between the patient, the professional, and the healthcare system, and includes barriers that belong to all three parts^(25)^. Some studies showed that patients with a greater number of comorbidities and a low functional status are least probable to participate in CR^(26,27)^. In our study, there was a positive correlation between cardiovascular risk and absenteeism (p=0.01; r=0.4), confirming what is said in the literature.

The variables studied may have an influence in the results for the practice of CR, but they cannot be considered determinant, due to the low coefficient of determination obtained in the statistical analysis for most of the variables.

Some qualitative studies investigated the beliefs of patients about CR. Participants and non-participants were investigated at various time points after an acute myocardial infarction (AMI) and participation in the CR programs. The non-participants commonly had misunderstandings as to rest and the perception of effort^(28)^. Patients also demonstrated a lack of knowledge as to the content of CR and the perception that CR would involve primarily physical exercises, which would then be, selectively, appropriate for patients considered previously fit^(29,30)^.

According to the Stanford CR Group, the improvement of compliance to exercise, diet, and medication, as well as a focus on habits, such as quitting smoking, requires measures on the part of the patient and approximation with the healthcare system. They assert that the use of cognitive and behavioral changes in face of health, and the strategies for communication, among which, motivational interviews and training sessions, serve to increase adherence to the CR programs^(31)^.

The motivational stage along the CR program presented with the double final score when compared to the initial motivational stage, which corresponds to an increase in motivation during the CR program.

The initiatives for continuous improvement in the system also increase the probability of the team reaching success in helping with individual behavioral changes in patients. CR programs offer a unique opportunity for healthcare professional that play a fundamental role in this support^(2)^.

One important multicenter study^(32)^ on evaluation of compliance to treatment demonstrated in the population studied that 89.9% of the patients presented with good adherence to the drug treatment, 72% adhered to the diet recommended, and 51% to the exercise recommendations. However, those who showed a sedentary lifestyle before the interventions presented with low adherence to the diet; a great proportion of elderly patient with comorbidities showed a tendency to continue with their sedentary habits, interrupting drug treatment and diet. These findings reinforce the fact that the use of CR programs, by means of a multidisciplinary team, may be critically important for changes of habits, especially in these populations.

We believe that mapping of the population participating in CR is fundamental for reaching greater adherence and positive results.

### Study limitations

It is important to point out that the data in this study are in reference to a very specific population, with a specific social/economic status and higher level of schooling, which may be related to our findings, primarily those related to the reasons for absences and cancelations due to travels and professional commitments. Another point to be reinforced is that our model of CR in which each professional is responsible for a small number of patients (up to four) does not allow extending these results to programs carried out with large numbers of patients in a single session.

## CONCLUSION

At our service, the patients presented with an increase in readiness to change over the course of the cardiovascular rehabilitation program. The greater faithful attendance was related to greater readiness in patients, with lower cardiovascular risks, and to the elderly, in which “travel/ work conflicts” was the primary barrier that led the patients to present with lower adherence.

Knowledge as to the primary barriers can help us in the application of effective strategies, seeking an increase in adherence to CR programs.

## Appendix 1 Items of the Cardiac Rehabilitation Barriers Scale

**Table:**

Items	I do not participate in a cardiovascular rehabilitation program, or if I do participate, I missed several sessions due to:
1	Distance (for example, the program is too far for me to go);
2	Cost (for example, fuel, parking, bus ticket);
3	Transportation problems (for example, I do not drive and have no one to take me; public transportation is inaccessible or inefficient);
4	Family responsibilities (for example, caring for grandchildren, sons and daughters, husband, housework);
5	Because I did not know about cardiac rehabilitation (for example, the physician did not tell me about this);
6	Because I do not need cardiac rehabilitation (for example, I feel good, my heart problem has already been treated, it is not serious);
7	Because I exercise at home or in my community;
8	Bad weather;
9	Because I find exercise to be tiring or painful;
10	Travels (for example, vacation, for work);
11	Because I have little time (for example, I am very busy, rehabilitation times are not convenient for me);
12	My work responsibilities;
13	Because I don't have much energy;
14	Other health problems that keep me from participating (specify: _____________________________);
15	Because I am too old;
16	Because my doctor did not think it would be necessary;
17	Because many people with heart problems do not engage in cardiac rehabilitation and they are fine;
18	Because I can control my heart problems;
19	Because I think I was referred, but the rehabilitation program did not contact me;
20	Because it took too long for me to be referred to the program and to initiate it;
21	Because I prefer to care for my health myself, not in a group;
22	Other reason(s) for not participating in a cardiac rehabilitation program: _________________________.

The scale described above was adapted with the addition of the twenty-second item, according to the scale validated for the Portuguese language by Ghisi et al.^(15)^.

## Appendix 2 Readiness to Change Scale

**Table:**

How important is it for you to make this change?			
	0…1…2…3…4…5…6…7…8…9…10		
	Not important	Extremely important	
How confident do you feel to make this change?			
	0…1…2…3…4…5…6…7…8…9…10		
	Not important	Extremely important	
According to the score attributed, we can infer the motivational stage of the client, according to the chart below:			
	Levels of readiness to change		
Not prepared	Insecure	Prepared	Changing
1…2…	3…4…5…	6…7…8…	9…10
Precontemplation	Contemplation	Preparation	Action

Translated and adapted from: Prochaska and DiClemente^(19)^.
